# Whole-Body Cryotherapy Decreases the Levels of Inflammatory, Oxidative Stress, and Atherosclerosis Plaque Markers in Male Patients with Active-Phase Ankylosing Spondylitis in the Absence of Classical Cardiovascular Risk Factors

**DOI:** 10.1155/2018/8592532

**Published:** 2018-02-01

**Authors:** Agata Stanek, Armand Cholewka, Tomasz Wielkoszyński, Ewa Romuk, Aleksander Sieroń

**Affiliations:** ^1^School of Medicine with the Division of Dentistry in Zabrze, Department of Internal Medicine, Angiology and Physical Medicine, Medical University of Silesia, Batorego Street 15, 41-902 Bytom, Poland; ^2^Department of Medical Physics, Chełkowski Institute of Physics, University of Silesia, 4 Uniwersytecka St., 40-007 Katowice, Poland; ^3^School of Medicine with the Division of Dentistry in Zabrze, Department of Biochemistry, Medical University of Silesia, Jordana 19 St., 41-808 Zabrze, Poland

## Abstract

**Objective:**

The aim of the study was to estimate the impact of whole-body cryotherapy (WBC) on cardiovascular risk factors in patients with ankylosing spondylitis (AS).

**Material and Methods:**

We investigated the effect of WBC with subsequent kinesiotherapy on markers of inflammation, oxidative stress, lipid profile, and atherosclerosis plaque in male AS patients (WBC group). To assess the disease activity, the BASDAI and BASFI were also calculated. The results from the WBC group were compared with results from the kinesiotherapy (KT) group.

**Results:**

The results showed that in the WBC group, the plasma hsCRP level decreased without change to the IL-6 level. The ICAM-1 level showed a decreasing tendency. The CER concentration, as well as the BASDAI and BASFI, decreased in both groups, but the index changes of disease activity were higher in the WBC than KT patients. Additionally, in the WBC group, we observed a decrease in oxidative stress markers, changes in the activity of some antioxidant enzymes and nonenzymatic antioxidant parameters. In both groups, the total cholesterol and LDL cholesterol, triglycerides, sCD40L, PAPP-A, and PLGF levels decreased, but the parameter changes were higher in the WBC group.

**Conclusion:**

WBC appears to be a useful method of atherosclerosis prevention in AS patients.

## 1. Introduction

Patients with ankylosing spondylitis (AS) have a higher risk of cardiovascular morbidity and mortality in comparison to the general population, which may be connected with the disease's activity, the functional and mobility limitations, structural damage, and inflammation [1, 2]. Even AS patients without concomitant classical cardiovascular risk factors yet, but in an active phase of the disease, are characterized by increased levels of oxidative stress, inflammatory states, higher serum concentrations of soluble CD40 ligand (sCD40L), and increased carotid intima-media thickness (IMT) in comparison to the general population. These factors may accelerate atherosclerosis in this group of patients [3, 4].

Fortunately, over the last several years, a revolution in the treatment of AS has taken place through the introduction of biological and disease-modifying antirheumatic drugs (DMARDs). Despite these advances, exercise and physiotherapy still play a very important role [5, 6].

A relatively new physiotherapeutic method used in the rheumatic disease treatment is whole-body cryotherapy (WBC), which is based on the therapeutic exposure of the entire human body to very low temperatures (below −100°C) for 120–180 seconds [7].

Recent studies have confirmed the anti-inflammatory, antianalgesic, and antioxidant effects of extremely low temperatures in athletes [8]. WBC procedures also have had a beneficial influence on lipid profiles in healthy subjects [9] and in obese people [10].

In addition, noticeably positive effects on the mental state [11] and antioxidant status of patients with multiple sclerosis [12] and seropositive rheumatoid arthritis [13] have been observed when low temperatures were applied to the entire body.

Little is still known about the role of WBC in the management of AS patients. So far, the studies have shown that WBC procedures in AS patients do not influence ejection fraction, late ventricular potentials, nor QT dispersion. However, they do have a beneficial effect on the adaptive processes of the vegetative nervous system in patients without a significant pathology in the circulatory system [14].

It has also been proved that in AS patients, WBC procedures with subsequent kinesiotherapy may improve BASDAI (Bath Ankylosing Spondylitis Diseases Activity Index) and BASFI (Bath Ankylosing Spondylitis Functional Index) and some spinal mobility parameters and help to decrease pain [15, 16].

In our preliminary study [17], we showed that WBC may also have a beneficial influence on some specific inflammatory parameters in AS patients.

In light of the above findings, the primary aim of the study was to assess the influence of WBC on cardiovascular risk factors in AS patients with active phase and without any concomitant classical cardiovascular risk factors.

## 2. Materials and Methods

### 2.1. Participants

The study protocol had been reviewed and approved by the Bioethical Committee of the Medical University of Silesia in Katowice (permission number: NN-6501-93/I/07), and all analyzed patients were informed about the trial and provided written consent for inclusion in the study. All clinical investigations were conducted according to the principles expressed in the Declaration of Helsinki (1964).

The study involved a total of 32 nonsmoking male patients with ankylosing spondylitis who were divided randomly by a physician into two groups with an allocation ratio 1 : 1. The first group consisted of 16 AS patients exposed to whole-body cryotherapy procedures with subsequent kinesiotherapy (WBC group, mean age 46.63 ± 1.5 years). The second group consisted of 16 AS patients exposed only to kinesiotherapy procedures (KT group, mean age 45.94 ± 1.24 years). There was no significant difference in the mean age, BMI, carotid IMT, BASDAI, BASFI, and comorbiding disorders and distribution of classical cardiovascular risk factors between these groups.

Computer-generated random numbers were sealed in sequentially numbered envelopes, and the group allocation was independent of the time and person delivering the treatment. The physician (main coordinator) who allocated the patients to groups had 32 envelopes, each containing a piece of paper marked with either group WBC or KT. The physician selected and opened each envelope in the presence of a physiotherapist to see the symbol and would then direct the subject to the corresponding group.

Male patients who successfully enrolled in the study had a definite diagnosis of AS, did not suffer from any other diseases, had no associated pathologies, and had an attending physician who did not apply disease-modifying antirheumatic drugs (DMARDs), biologic agents, or steroids. The AS patients were treated with doses of nonsteroidal anti-inflammatory drugs (NSAIDs), which were not altered within one month before the beginning of the study and during it. All the patients included in the trial fulfilled the modified New York Criteria for definite diagnosis of AS, which serves as the basis for the ASAS/EULAR recommendations [18]. The final selection for the study included only HLA B27-positive patients, who exhibited II and III radiographic grades of sacroiliac joint disease and attended a consulting unit in a health resort in the period of subsidence of acute clinical symptoms, in order to qualify for sanatorium treatment (physiotherapy). The demographic data of the subjects is shown in Table 1.

The patients from both groups were asked to abstain from alcohol, drugs and any immunomodulators, immunostimulators, hormones, vitamins, minerals, or other substances with antioxidant properties for 4 weeks before the study. All the patients were also asked to refrain from the consumption of caffeine 12 hours prior to laboratory analyses. The diet of the patients was not modified.

Before the study, each patient was examined by a physician to exclude any coexisting diseases as well as any contraindications for WBC procedures. Prior to the study, a resting electrocardiogram was performed on all the patients, and before each session of cryotherapy, the blood pressure was measured for each patient.

### 2.2. Whole-Body Cryotherapy and Kinesiotherapy Procedures

Depending on the group, the AS patients were exposed either to a cycle of WBC procedures lasting 3 minutes a day with a subsequent 60-minute session of kinesiotherapy or to a 60-minute session of kinesiotherapy only, for 10 consecutive days excluding the weekend.

The WBC procedures were performed in a cryochamber with cold retention and cooled by synthetic liquid air (produced by Metrum Cryoflex, Poland), which consists of two compartments: the antechamber and the proper chamber, which were connected by a door. In the trial, the temperature in the antechamber was −60°C, whereas in the proper chamber, it reached −120°C. After a 30-second adaptation process in the antechamber, the patients were exposed to cryogenic temperatures in the proper chamber for 3 minutes. During the WBC procedure, all the patients were dressed in swimsuits, cotton socks and gloves, and wooden shoes and their mouths and noses were protected by surgical masks and their ears by ear protectors. All jewelry, glasses, and contact lenses were removed before entry into the chamber. During the WBC procedure, the patients were walking round the chamber without touching each other.

Immediately after leaving the cryogenic chamber and changing into track suits and trainers, the AS patients underwent kinesiotherapy lasting one hour. The program of kinesiotherapy was the same for all the patients in both groups. Kinesiotherapy procedures included range-of-motion exercises of the spine and major joints (including the ankle, knee, hip, wrist, elbow, and shoulder). Chest expansion and breathing exercises were also included. Apart from range-of-motion exercise, the AS patients received strengthening exercises of the muscles of the major joints (including the ankle, knee, hip, wrist, elbow, shoulder, thoracolumbar spine, and cervical spine) as well as aerobic exercise (including cycling and fast walking). All the exercises were carried out under the supervision of physical therapists.

All the patients completed the study and no complications or side effects related to the WBC procedures were observed.

### 2.3. Blood Sample Collection

Blood samples of all the subjects were collected in the morning before the first meal. Samples of whole blood (5 ml) were drawn from the basilic vein of each subject and then collected into tubes containing ethylenediaminetetraacetic acid (Sarstedt, S-Monovette with 1.6 mg/ml EDTA-K_3_) and into tubes with a clot activator (Sarstedt, S-Monovette). The blood samples were centrifuged (10 min, 900*g* at 4°C), and then the plasma and serum were immediately separated and stored at the temperature of −75°C, until biochemical analyses could be performed. In turn, the red blood cells retained from the removal of EDTA plasma were rinsed with isotonic salt solution and then 10% of the hemolysates were prepared for further analyses. The hemoglobin concentration in the hemolysates was determined by the standard cyanmethemoglobin method. The inter- and intra-assay coefficients of variations (CV) were 1.1% and 2.4%, respectively.

### 2.4. Biochemical Analyses

#### 2.4.1. Determination of Inflammatory-State Parameters

High-sensitivity C-reactive protein (hs-CRP) concentration in the serum was determined by the latex immunoturbidimetric method (BioSystems, Spain) and expressed in mg/l. The inter- and intra-assay coefficients of variations (CV) were 2.3% and 5.5%, respectively.

The serum ceruloplasmin (CER) oxidase activity was measured using the p-phenylenediamine kinetic method by Richterich [19] and expressed in mg/dl after a calibration with pure ceruloplasmin isolated from a healthy donor serum pool. The inter- and intra-assay coefficients of variations (CV) were 3.1% and 6.1%, respectively.

The plasma interleukin 6 (IL-6) and soluble intercellular adhesion molecule-1 (sICAM-1) concentrations were determined using the ELISA method from R&D Systems (USA). The concentrations of IL-6 and sICAM-1 were expressed in pg/ml and ng/ml. The inter- and intra-assay coefficients of variations (CV) were 5.1% and 8.8%, respectively, for IL-6 and 4.8% and 9.1%, respectively, for sICAM-1.

#### 2.4.2. Oxidative Stress Marker Analyses

*(1) Determination of Lipid Peroxidation Products, Total Oxidative Status, and Oxidative Stress Index*. The intensity of lipid peroxidation in the plasma and the erythrocytes was measured spectrofluorimetrically as thiobarbituric acid-reactive substances (TBARS) according to Ohkawa et al. [20]. The TBARS concentrations were expressed as malondialdehyde (MDA) equivalents in *μ*mol/l in plasma or in nmol/gHb in erythrocytes. The inter- and intra-assay coefficients of variations (CV) were 2.1% and 8.3%, respectively.

The serum concentrations of oxidized low-density lipoprotein (ox-LDL) and antibodies to ox-LDL (ab-ox-LDL) were measured with the use of ELISA kits (Biomedica, Poland). The ox-LDL and the ab-ox-LDL concentrations were expressed in ng/ml and mU/ml, respectively. The inter- and intra-assay coefficients of variations (CV) for ox-LDL were 5.8% and 9.4%, respectively, and −4.1% and 8.7% for ab-ox-LDL, respectively.

The serum total oxidant status (TOS) was determined with the method described by Erel [21] and expressed in *μ*mol/l. The inter- and intra-assay coefficients of variations (CV) were 2.2% and 6.4%, respectively.

The oxidative stress index (OSI), an indicator of the degree of oxidative stress, was expressed as the ratio of total oxidant status (TOS) to total antioxidant capacity (FRAP) in arbitrary units [22].

*(2) Determination of Activity of Antioxidant Enzymes*. The plasma and erythrocytes superoxide dismutase (SOD - E.C.1.15.1.1) activity was determined by the Oyanagui method [23]. Enzymatic activity was expressed in nitrite unit (NU) in each mg of hemoglobin (Hb) or ml of blood plasma. One nitrite unit (1 NU) means a 50% inhibition of nitrite ion production by SOD in this method. SOD isoenzymes (SOD-Mn and SOD-ZnCu) were measured using potassium cyanide as the inhibitor of the SOD-ZnCu isoenzyme. The inter- and intra-assay coefficients of variations (CV) were 2.8% and 5.4%, respectively.

The catalase (CAT - E.C.1.11.1.6.) activity in erythrocytes was measured by the Aebi [24] kinetic method and expressed in IU/mgHb. The inter- and intra-assay coefficients of variations (CV) were 2.6% and 6.1%, respectively.

The erythrocyte glutathione peroxidase (GPx - E.C.1.11.1.9.) activity was assayed by Paglia and Valentine's kinetic method [25], with t-butyl peroxide as a substrate and expressed as micromoles of NADPH oxidized per minute and normalized to one gram of hemoglobin (IU/gHb). The inter- and intra-assay coefficients of variations (CV) were 3.4% and 7.5%, respectively.

The activity of glutathione reductase in erythrocytes (GR - E.C.1.6.4.2) was assayed by Richterich's kinetic method [19], expressed as micromoles of NADPH utilized per minute and normalized to one gram of hemoglobin (IU/gHb). The inter- and intra-assay coefficients of variations (CV) were 2.1% and 5.8%, respectively.

*(3) Determination of Nonenzymatic Antioxidant Status*. The total antioxidant capacity of plasma was measured as the ferric-reducing ability of plasma (FRAP) according to Benzie and Strain [26] and calibrated using Trolox and expressed in (*μ*mol/l). The inter- and intra-assay coefficients of variations (CV) were 1.1% and 3.8%, respectively.

The serum concentration of protein sulfhydryl (PSH) was determined by Koster's method [27], using dithionitrobenzoic acid (DTNB) and expressed in (*μ*mol/l). The inter- and intra-assay coefficients of variations (CV) were 2.6% and 5.4%, respectively.

The serum concentration of uric acid (UA) was determined by a uricase-peroxidase method [28] on the Cobas Integra 400 plus analyzer and expressed as (mg/dl). The inter- and intra-assay coefficients of variations (CV) were 1.4% and 4.4%, respectively.

#### 2.4.3. Determination of Lipid Profile

The total, HDL, and LDL cholesterol (T-Chol, HDL-Chol, and LDL-Chol, resp.) and triglyceride (TG) concentrations in serum were estimated using routine techniques (Cobas Integra 400 plus analyzer, Roche Diagnostics, Mannheim, Germany). The concentrations were expressed in (mg/dl). The inter- and intra-assay coefficients of variations (CV) were 2.8% and 5.4%, respectively, for T-Chol; 3.2% and 5.4%, respectively, for HDL-Chol; 2.6% and 6.5%, respectively, for LDL-Chol; and 2.5% and 7.6%, respectively, for TG. The triglyceride/HDL cholesterol (TG/HDL) ratio was calculated.

#### 2.4.4. Determination of Atherosclerosis Plaque Instability Markers and Atherosclerosis Plaque Markers

Serum pregnancy-associated plasma protein-A (PAPP-A), soluble CD40 ligand (sCD40L), and placental growth factor (PLGF) concentrations were assayed by ELISA methods with DRG Instruments GmbH (Germany). The PAPP-A and sCD40L concentrations were expressed in ng/ml and the PLGF concentration in pg/ml. The inter- and intra-assay coefficients of variations (CV) were 6.8% and 10.2%, respectively, for PAPPA-A; 5.1% and 9.4%, respectively, for sCD40L; and 6.2% and 12.1%, respectively, for PLGF.

### 2.5. Assay of Activity of Ankylosing Spondylitis

The activity of ankylosing spondylitis was measured by the Bath Ankylosing Spondylitis Diseases Activity Index (BASDAI) and the Bath Ankylosing Spondylitis Functional Index (BASFI).

The BASDAI has six questions related to fatigue, back pain, peripheral pain, peripheral swelling, local tenderness, and morning stiffness (degree and length). Other than the issues relating to morning stiffness, all questions were scored from 0 (none) to 10 (very severe) using a visual analogue scale (VAS). The sum was calculated as the mean of two morning stiffness issues and the four remaining issues [29].

The BASFI is the mean score of ten questions addressing functional limitations and the level of physical activity at home and work, assessed on VAS scales (0 = easy, 10 = impossible) [30].

### 2.6. Assay of Intima-Media Thickness

A high-resolution Doppler ultrasonography was performed with a Logic-5 device with a high-frequency (11 MHz, 15 MHz) linear probe. The sonographer was an angiologist who was unaware of subject's clinical state. The measurement of intima-media thickness (IMT) was performed in the right and left common carotid arteries, and the average of the 2 measurements was calculated. The IMT was expressed in mm.

### 2.7. Statistical Analyses

Statistical analyses were undertaken using the statistical package of Statistica 10 Pl software. For each parameter, the indicators of the descriptive statistics were determined (mean value and standard deviation (SD)). The normality of the data distribution was checked using the Shapiro-Wilk test, while the homogeneity of the variance was checked by applying Levene's test. In order to compare the differences between the groups, an independent sample Student *t*-test was used or alternatively the Mann–Whitney *U* test. In the case of dependent samples, the Student *t*-test was used or alternatively the Wilcoxon test. Correlations between particular parameters were statistically verified by means of Spearman's nonparametric correlation test. Differences at the significance level of *P* < 0.05 were considered as statistically significant.

## 3. Results

### 3.1. Inflammatory-State Parameters, BASDAI, and BASFI

In the WBC group of AS patients, who underwent a ten-day-long cycle of WBC procedures with subsequent kinesiotherapy, it was found that after the completion of the treatment, the levels of hsCRP and CER decreased significantly. In the case of hsCRP, the difference prior to post treatment values in the WBC group was significantly higher in comparison to those in the KT group patients. Also, in the WBC group, the level of sICAM-1 showed a decreasing trend. Moreover, after the completion of the WBC cycle, the level of sICAM-1 was significantly lower in comparison to the KT group. But the level of IL-6 did not change significantly in the WBC group with subsequent kinesiotherapy after the completion of treatment.

After the completion of treatment, only the level of CER decreased significantly from the estimated inflammatory parameters in AS patients from the KT group who underwent a cycle of kinesiotherapy only, without being preceded by WBC procedures. The levels of hsCRP and sICAM-1 did not change significantly in the KT group. Also, as in the WBC group, no statistically significant changes in the level of IL-6 were observed in the KT group.

In turn, the BASDAI and BASFI decreased significantly in both groups, but in the WBC group with subsequent kinesiotherapy after the completion of the treatment, the decrease of these parameters was significantly higher in comparison to that in the KT group. Moreover, only in the WBC group after the completion of the treatment, the value of both BASDAI and BASFI was below 4 (inactive phase of AS disease) (Table 2).

### 3.2. Oxidative Stress

We observed that patients in the WBC group had, after the completion of the treatment, a statistically significant decrease in erythrocyte levels of MDA, serum anti-ox-LDL ab, serum TOS, and value of OSI in comparison to initial values. What is more, the differences of these parameters prior to post treatment values in the WBC group were significantly higher in comparison to the KT group. The levels of plasma MDA and serum ox-LDL did not change significantly in the WBC group. In turn, in the KT group, no significant changes in the levels of plasma and erythrocyte MDA, serum ox-LDL, serum anti-ox-LDL ab, and serum TOS and OSI were observed after the completion of the treatment, in comparison to the initial values before the beginning of the kinesiotherapy cycle (Table 3).

In the WBC group patients, we observed a statistically significant decrease in erythrocyte activity of GPx after the completion of a cycle of cryotherapy procedures with subsequent kinesiotherapy. However, the activity of plasma and erythrocyte total SOD, plasma SOD-Mn, plasma SOD-CuZn, erythrocyte CAT, and GR did not change significantly in the WBC group after treatment. But in the WBC group, the activity of plasma SOD-Mn after treatment was significantly higher in comparison to the KT group. In turn, in the KT group, the activity of erythrocyte total SOD, GPx, and GR decreased significantly after treatment in comparison to the WBC group. Additionally, the activity of plasma SOD-CuZn showed also a decreased tendency in the KT group. Similarly as in the WBC group patients, the activity of plasma total SOD and erythrocyte CAT did not change significantly in the KT group after treatment (Table 4).

What is more, in the WBC group, the parameters of nonenzymatic antioxidants, FRAP values, and UA concentration increased significantly after treatment. The levels of those parameters were significantly higher in the WBC group in comparison to the KT group after the completion of the treatment. The level of PSH did not change significantly in the WBC group after treatment. In turn, in the KT group, the FRAP values and PSH level decreased significantly, but the level of UA did not change significantly after treatment (Table 5).

### 3.3. Markers of Lipid Profile, Atherosclerosis Plaque, and Atherosclerosis Plaque Instability

The levels of T-Chol, LDL, TG, sCD40L, PLGF, and PAPP-A decreased significantly after treatment in both groups, but the differences prior to post treatment values in the WBC group were significantly higher in comparison to the KT group, except for T-Chol. But the TG difference prior to post treatment values in the WBC group was higher in comparison to the KT group. The level of HDL-Chol did not change significantly in both groups. The TG/HDL ratio showed a decreasing tendency in the WBC group in comparison to the KT group (Table 6).

### 3.4. Significant Relationships among the Estimated Parameters in AS Patients Who Underwent WBC Procedures

After treatment, we noticed significant relationships in the WBC group between changes of serum hsCRP concentration and erythrocyte MDA concentration (*r* = 0.6). Also, a positive correlation between serum hsCRP change and plasma FRAP activity change (*r* = 0.6) was observed. Additionally, a negative correlation between serum hsCRP concentration and plasma SOD-CuZn activity was found (*r* = −0.62). In the case of the analysis of serum oxLDL-ab, we observed a negative correlation with CAT and SOD activities in erythrocytes (*r* coefficients: −0.51 and −0.53, resp.). Furthermore, the ratio of TG/HDL was positively correlated with the PLGF serum concentration after WBC procedures (*r* = 0.58). We also observed a positive correlation between plasma concentrations of sICAM-1 and MDA (*r* = 0.66) in the WBC group after treatment. In the case of erythrocyte GPx activity in AS patients who underwent WBC procedures with subsequent kinesiotherapy, a positive correlation with plasma PSH (*r* = 0.54) was visible and a negative correlation was found with plasma MDA concentration. All the correlations mentioned above were significant (*p* < 0.05).

## 4. Discussion

In our study, we observed that, after the completion of the treatment, the WBC group of AS patients who underwent a ten-day-long cycle of WBC procedures with subsequent kinesiotherapy had significantly decreased levels of hsCRP and CER. The level of sICAM-1 showed a decreasing trend in the WBC group. But the level of IL-6 did not change significantly.

The results of the inflammatory parameters in this study are consistent with our previous preliminary study [17], in which AS patients who underwent WBC procedures were observed to have a decrease in CRP, fibrinogen, mucoprotein, and sICAM levels.

However, in another study [31], the authors have observed a decrease in TNF-*α* and an increase in IL-6 in tennis players after a 5-day exposure to WBC twice a day.

Banfi et al. [32] have also confirmed that a decreased level of sICAM-1 is induced by WBC treatment and is linked to an anti-inflammatory response. In another paper, Pournot et al. [33] have found that WBC (−110°C) decreased IL-1*β* and CRP levels and increased the IL-1ra level after intense exercise. But the levels of TNF-*α*, IL-10, and IL-6 remained unchanged. Similarly, in our study, we did not observe any changes in serum IL-6 in AS patients who underwent WBC.

In the present study, we also saw a significant decrease in the BASDAI and BASFI after the completion of the WBC treatment in a cryochamber with cold retention. Similar results were observed in a closed cryochamber of a type called “Wrocławski”, cooled by liquid nitrogen [15]. In the both studies, after the completion of a cycle consisting of ten daily 3-minute-long WBC procedures with subsequent kinesiotherapy (−120°C, with a weekend break), the BASDAI and BASFI decreased below 4. This indicates that the AS disease entered an inactive phase after the completion of treatment. Our results are also consistent with a study [16], in which the AS patients underwent 8 daily WBC procedures (−110°C, 3 minutes).

There are not many reports on the impact of WBC on the prooxidant-antioxidant balance. It has been noticed that WBC procedures may have a beneficial influence on antioxidant status. In the study performed by Dugué et al. [34], a significant increase has been seen in the TAS value in healthy men at the end of a cycle of 45 procedures of WBC (−110°C, 2 minutes, coolant liquid nitrogen) performed three times a week. In another study, Miller et al. [12] have noticed an increase in total antioxidant status, SOD activity, and uric acid level in the plasma of multiple sclerosis patients who underwent WBC treatment (−110° temperature, daily 10 procedures with weekend break, coolant medium liquid nitrogen). What is more, WBC was advocated to possibly enhance antioxidant capacities and, thus, counteract the exercise-induced reactive oxygen species production [12].

However, in a different study [13], patients with seropositive rheumatoid were observed by the authors to have only a short-term increase in TRAP during the first treatment session of WBC (−110°C, three times daily for 7 consecutive days) and the cold treatment did not cause any significant oxidative stress or adaptation.

In our study, we observed a significant decrease in oxidative stress, which may also be linked to the decrease in systemic inflammation in AS patients who underwent WBC treatment. After treatment, in the WBC group, we observed positive correlations between plasma concentrations of sICAM-1 and MDA as well as serum hsCRP and erythrocyte MDA concentrations. In addition, negative correlations between serum hsCRP concentration and plasma SOD-CuZn activity were found.

Furthermore, we observed the similar results in healthy subjects who underwent WBC procedures performed in a cryochamber with cold retention [35].

The differences in the results of various studies may be related to the type of cryochamber being used and the coolant medium, in addition to the time of exposure to cryogenic temperatures.

Only a few papers have estimated the impact of WBC on lipid profile. In rats exposed to WBC for 5 or 10 days, HDL and LDL cholesterol fraction decreased and total cholesterol levels in animals subjected to −60°C sessions for 10 days remained unchanged. The authors have also observed an increase in triglycerides in the blood serum of animals subjected to cryostimulation compared to control. A decrease in HDL cholesterol in rats after cryostimulation can be explained by the fact that HDL is the main fraction transporting cholesterol in rats, while in humans, most cholesterol is found in low-density lipoproteins [36].

In another study [9], the authors have observed reducing T-Chol, LDL-Chol, and TG and increasing HDL-Chol after 20 sessions of WBC in healthy men, but after 10 sessions of WBC, only LDL-Chol decreased, while a simultaneous HDL-Chol increase was observed in healthy men (cryogenic temperature −130°C).

In another study by these authors [14], a significant decrease in the level of LDL-Chol and TG has been observed, with a slight increase in high-density lipoprotein concentration after WBC treatment, including two cryostimulation treatments of 20 daily sessions in the second and the last month of intervention, without diet modification in obese subjects.

In our study, we also observed a significant decrease in T-Chol, LDL-Chol, and TG. But the HDL-Chol level did not change after completing WBC procedures in the AS patients. What is more, in our study, we observed a significant decrease in the levels of sCD40, PAPP-A, and PLGF. Additionally, in the present study, the ratio of TG/HDL was positively correlated with the PLGF serum concentration after WBC procedures. The impact of WBC on these markers in AS patients has been estimated for the first time.

A significant decrease in lipid profile, atherosclerotic plaque and oxidative stress, and inflammatory parameters, as well as a reduction in the proportion of TG cholesterol to HDL cholesterol (TG/HDL ratio), seems beneficial enough to consider WBC treatment as a useful method for atherosclerosis prevention in AS patients.

The present study has some limitations. First, the study did not provide long-term follow-up (at least 3 months), and thus, we do not know how long the beneficial effect of WBC with subsequent kinesiotherapy would be maintained after the completion of a WBC cycle. Second, the cycle of WBC with subsequent kinesiotherapy consisted of only ten procedures. A greater number of procedures (e.g., 20–30) could probably increase the treatment effect. Third, the study should involve a larger number of AS patients.

## 5. General Conclusion

Whole-body cryotherapy with subsequent kinesiotherapy facilitates a decrease in oxidative stress, lipid profile, atherosclerosis plaque, and its instability, as well as inflammatory parameters, and appears to be a useful method of atherosclerosis prevention in AS patients.

## Figures and Tables

**Table 1:** Demographic data of the study subjects.

Characteristic	WBC group (*n* = 16)	Kinesiotherapy group (*n* = 16)	*P* value
Age (years), mean (SD)	46.63 ± 1.5	45.94 ± 1.24	0.114
Sex (M/F)	16/0	16/0	—
BMI (kg/m^2^), mean (SD)	24.24 ± 4.4	23.76 ± 6.8	0.880
BASDAI	5.43 ± 1.61	5.28 ± 1.71	0.720
BASFI	5.20 ± 2.29	5.01 ± 2.06	1.00
Carotid IMT (mm)	1.1 ± 0.13	1.0 ± 0.14	0.925
Smoking (yes/no)	0/16	0/16	—
Medication			
NSAID (yes/no)	16/0	16/0	—
DMARD (yes/no)	0/16	0/16	—
Biological agents (yes/no)	0/16	0/16	—

SD: standard deviation; BMI: body mass index; BASDAI: the Bath Ankylosing Spondylitis Diseases Activity Index; BASFI: the Bath Ankylosing Spondylitis Functional Index; IMT: intima-media thickness; NSAID: nonsteroidal anti-inflammatory drug; DMARD: disease-modifying antirheumatic drug.

**Table 2:** Levels of inflammatory parameters as well as the value of BASDAI and BASFI (mean value ± standard deviation (SD)) in AS patients before and after the completion of a cycle of ten whole-body cryotherapy procedures with subsequent kinesiotherapy (WBC group) or a cycle of ten kinesiotherapy procedures only (KT group), with statistical analyses. (p): plasma; (s): serum; Δ: difference prior to post treatment.

Parameters		WBC group	KT group	*P*
hsCRP (s) (mg/l)	Before	13.5 ± 16.3	13.9 ± 15.2	0.942
	After	9.2 ± 15.3	13.6 ± 16.2	0.438
	^∗^ *P*	**0.002**	0.623	
	Δ	−4.24 ± 5.68	−0.27 ± 3.25	**0.023**

CER (s) (mg/dl)	Before	62.83 ± 12.61	67.57 ± 12.60	0.296
	After	51.32 ± 10.74	53.51 ± 14.26	0.628
	^∗^ *P*	**0.006**	**0.003**	
	Δ	−11.51 ± 16.6	−14.06 ± 14.47	0.646

IL-6 (p) (pg/ml)	Before	41.6 ± 8.86	41.8 ± 10.5	0.957
	After	36.6 ± 7.89	41.0 ± 10.4	0.191
	^∗^ *P*	0.121	0.301	0.216
	Δ	−4.94 ± 11.9	−0.74 ± 5.71	

sICAM-1 (p) (ng/ml)	Before	79.0 ± 15.5	84.3 ± 21.9	0.432
	After	69.2 ± 14.2	83.9 ± 20.0	**0.023**
	^∗^ *P*	0.088	0.642	
	Δ	−9.84 ± 23.0	−0.41 ± 16.1	0.191

BASDAI	Before	5.43 ± 1.61	5.28 ± 1.71	0.720
	After	3.29 ± 0.91	4.53 ± 1.62	**<0.05**
	^∗^ *P*	**<0.001**	**<0.001**	
	Δ	−2.14 ± 1.23	−0.74 ± 0.38	**0.001**

BASFI	Before	5.20 ± 2.29	5.01 ± 2.06	1.00
	After	3.81 ± 2.20	4.35 ± 2.23	0.497
	^∗^ *P*	**<0.001**	**<0.001**	
	Δ	−1.39 ± 1.03	−0.66 ± 0.39	**<0.01**

*P*: statistical significance of differences between both groups of patients; ^∗^*P*: statistical significance of differences between values before and after treatment in particular groups of patients.

**Table 3:** Levels of lipid peroxidation parameters, total oxidative status (TOS), and oxidative stress index (OSI) (mean value ± standard deviation (SD)) in AS patients before and after the completion of a cycle of ten whole-body cryotherapy procedures with subsequent kinesiotherapy (WBC group) or a cycle of ten kinesiotherapy procedures only (KT group), with statistical analyses. (p): plasma; (s): serum; (e): erythrocyte lysates; Δ: difference prior to post treatment.

Parameters		WBC group	KT group	*P*
MDA (p) (*μ*mol/l)	Before	2.54 ± 0.52	2.32 ± 0.60	0.272
	After	2.30 ± 0.75	2.41 ± 0.83	0.715
	^∗^ *P*	0.278	0.959	
	Δ	−0.24 ± 0.81	0.09 ± 1.04	0.331

MDA (e) (nmol/gHb)	Before	0.17 ± 0.04	0.18 ± 0.02	0.418
	After	0.15 ± 0.03	0.18 ± 0.04	**0.007**
	^∗^ *P*	**0.013**	0.642	
	Δ	−0.02 ± 0.03	0.00 ± 0.04	**0.043**

ox-LDL (s) (ng/ml)	Before	249 ± 77.6	298 ± 122	0.191
	After	223 ± 100	288 ± 133	0.132
	^∗^ *P*	0.301	0.84	
	Δ	−25.9 ± 123	−9.6 ± 149	0.738

Anti-oxLDL ab (s) (mU/ml)	Before	465 ± 209	571 ± 426	0.382
	After	347 ± 139	490 ± 316	0.111
	^∗^ *P*	**0.013**	0.379	
	Δ	−118 ± 178	−80.5 ± 323	0.687

TOS (s) (*μ*mol/l)	Before	26.54 ± 4.45	23.94 ± 11.60	0.414
	After	12.09 ± 2.55	24.41 ± 6.24	**<0.001**
	^∗^ *P*	**<0.001**	0.605	
	Δ	−14.45 ± 4.83	0.46 ± 9.11	**<0.001**

OSI (p/s) (arbitrary unit)	Before	24.10 ± 15.94	18.87 ± 11.30	0.294
	After	8.20 ± 6.76	23.65 ± 15.68	**0.002**
	^∗^ *P*	**0.003**	0.301	
	Δ	−15.90 ± 16.82	4.78 ± 13.88	**0.001**

*P*: statistical significance of differences between both groups of patients; ^∗^*P*: statistical significance of differences between values before and after treatment in particular groups of patients.

**Table 4:** Activities of antioxidant enzymes (mean value ± standard deviation (SD)) in AS patients before and after the completion of a cycle of ten whole-body cryotherapy procedures with subsequent kinesiotherapy (WBC group) or a cycle of ten kinesiotherapy procedures only (KT group), with statistical analyses. (p): plasma; (e): erythrocyte lysates; Δ: difference prior to post treatment.

Parameters		WBC group	KT group	*P*
Total SOD (p) (NU/ml)	Before	13.4 ± 2.13	12.3 ± 1.85	0.145
	After	12.1 ± 1.88	1.7 ± 2.49	0.632
	^∗^ *P*	0.233	0.301	
	Δ	−1.28 ± 3.13	−0.60 ± 2.65	0.512

SOD-Mn (p) (NU/ml)	Before	5.37 ± 2.75	4.56 ± 1.86	0.336
	After	6.27 ± 0.99	5.02 ± 1.64	**0.015**
	^∗^ *P*	0.163	0.642	
	Δ	0.90 ± 2.80	0.46 ± 2.46	0.642

SOD-CuZn (p) (NU/ml)	Before	8.09 ± 2.74	7.80 ± 2.21	0.749
	After	7.15 ± 1.32	7.05 ± 3.09	0.902
	^∗^ *P*	0.326	0.063	
	Δ	−0.93 ± 2.77	−0.75 ± 2.72	0.854

Total SOD (e) (NU/mgHb)	Before	85.5 ± 17.3	128.0 ± 11.2	**<0.001**
	After	90.5 ± 11.9	111.0 ± 15.6	**<0.001**
	^∗^ *P*	0.438	**0.001**	
	Δ	5.02 ± 17.3	−17.1 ± 11.8	**<0.001**

CAT (e) (IU/mgHb)	Before	385.0 ± 70.3	425.0 ± 53.6	0.084
	After	375.0 ± 58.3	412.0 ± 58.6	0.088
	^∗^ *P*	0.535	0.352	
	Δ	−9.9 ± 57.0	−13.0 ± 54.0	0.876

GPx (e) (IU/gHb)	Before	31.2 ± 4.90	29.9 ± 2.84	0.363
	After	29.1 ± 2.97	20.4 ± 5.05	<0.001
	^∗^ *P*	**0.039**	**0.001**	
	Δ	−2.09 ± 3.61	−9.49 ± 6.74	0.001

GR (e) (IU/gHb)	Before	1.72 ± 0.56	2.07 ± 0.52	0.043
	After	1.54 ± 0.60	1.65 ± 0.59	0.078
	^∗^ *P*	0.469	**0.002**	
	Δ	−0.18 ± 0.80	−0.42 ± 0.41	0.622

*P*: statistical significance of differences between both groups of patients; ^∗^*P*: statistical significance of differences between values before and after treatment in particular groups of subjects.

**Table 5:** Levels of nonenzymatic antioxidants (mean value ± standard deviation (SD)) in AS patients before and after the completion of a cycle of ten whole-body cryotherapy procedures with subsequent kinesiotherapy (WBC group) or a cycle of ten kinesiotherapy procedures only (KT group), with statistical analyses. (p): plasma; (s): serum; Δ: difference prior to post treatment.

Parameters		WBC group	KT group	*P*
FRAP (*μ*mol/l)	Before	587.1 ± 58.3	550.0 ± 91.3	0.183
	After	636.1 ± 62.3	499.3 ± 74.6	**<0.001**
	^∗^ *P*	**0.010**	**0.001**	
	Δ	49.0 ± 31.7	−50.8 ± 39.4	**<0.001**

PSH (s) (*μ*mol/l)	Before	402.6 ± 91.7	393.2 ± 90.0	0.772
	After	392.6 ± 87.4	364.7 ± 28.4	0.239
	^∗^ *P*	0.836	**0.017**	
	Δ	−9.9 ± 108.1	−28.5 ± 92.6	0.605

UA (s) (mg/dl)	Before	5.40 ± 1.39	4.34 ± 1.15	**0.025**
	After	6.62 ± 2.07	4.61 ± 1.25	**0.003**
	^∗^ *P*	**0.011**	0.196	
	Δ	1.22 ± 1.70	0.27 ± 0.70	0.052

*P*: statistical significance of differences between both groups of patients; ^∗^*P*: statistical significance of differences between values before and after treatment in particular groups of patients.

**Table 6:** Levels of lipid profile parameters, atherosclerosis plaque markers, and atherosclerosis plaque instability and values of TG/HDL ratio (mean value ± standard deviation (SD)) in AS patients before and after the completion of a cycle of ten whole-body cryotherapy procedures with subsequent kinesiotherapy (WBC group) or a cycle of ten kinesiotherapy procedures only (KT group), with statistical analyses. (p): plasma; (s): serum; (e): erythrocyte lysates; Δ: difference prior to post treatment.

Parameters		WBC group	KT group	*P*
T-Chol (s) (mg/dl)	Before	221.3 ± 39.17	200.33 ± 21.33	0.074
	After	202.40 ± 24.40	190.70 ± 22.57	0.51
	^∗^ *P*	**0.0006**	**0.04**	
	Δ	−18.90 ± 20.54	−9.63 ± 18.38	0.20

LDL-Chol (s) (mg/dl)	Before	125.2 ± 32.6	145.3 ± 28.3	0.073
	After	93.3 ± 36.9	132.4 ± 24.7	**0.002**
	^∗^ *P*	**<0.001**	**0.005**	
	Δ	−31.9 ± 28.6	−12.9 ± 15.1	**0.027**

HDL-Chol (s) (mg/dl)	Before	50.5 ± 14.1	58.0 ± 18.0	0.198
	After	47.0 ± 9.0	56.4 ± 18.2	0.078
	^∗^ *P*	0.079	0.109	
	Δ	−3.5 ± 9.1	−1.7 ± 10.1	0.590

TG (s) (mg/dl)	Before	185.1 ± 18.9	178.6 ± 15.9	0.299
	After	156.7 ± 11.2	165.2 ± 20.4	0.158
	^∗^ *P*	**0.001**	**0.001**	
	Δ	−28.4 ± 22.4	−13.4 ± 19.7	0.053

TG/HDL ratio	Before	3.95 ± 1.18	3.32 ± 0.96	0.150
	After	3.44 ± 0.60	3.18 ± 0.99	0.320
	^∗^ *P*	0.055	0.250	
	Δ	−0.51 ± 0.92	−0.14 ± 0.43	0.190

sCD40L(s) (mg/ml)	Before	9.21 ± 3.88	7.25 ± 2.20	0.180
	After	5.01 ± 2.55	5.85 ± 2.06	0.171
	^∗^ *P*	**0.0004**	**0.006**	
	Δ	−4.19 ± 2.17	−1.4 ± 1.78	**0.0001**

PLGF(s) (pg/ml)	Before	30.17 ± 10.23	21.69 ± 3.54	0.007
	After	19.32 ± 5.53	18.31 ± 2.91	0.641
	^∗^ *P*	**0.001**	**0.004**	
	Δ	−10.84 ± 7.05	−3.38 ± 2.13	**0.0001**

PAPP-A (s) (ng/ml)	Before	17.74 ± 7.78	14.48 ± 4.52	0.162
	After	11.24 ± 3.12	11.79 ± 3.72	0.920
	^∗^ *P*	**0.0004**	**0.003**	
	Δ	−6.51 ± 8.40	−2.69 ± 3.65	**0.008**

*P*: statistical significance of differences between both groups of patients; ^∗^*P*: statistical significance of differences between values before and after treatment in particular groups of patients.
